# A dimensional approach to care: implementing a stepped model for ICD-11 personality disorders

**DOI:** 10.1192/j.eurpsy.2026.10316

**Published:** 2026-07-31

**Authors:** F. G. Di Leone

**Affiliations:** Sahlgrenska University Hospital, Gothenburg, Sweden

## Abstract

**Abstract:**

The ICD-11 introduces a dimensional, severity-based approach to personality disorder (PD) diagnosis, moving away from categorical typologies. While generally well received by clinicians and service providers, concrete examples of its implementation in clinical practice remain limited.

This presentation draws on an ongoing study examining the learnability and acceptability of the ICD-11 model across clinical and training settings in Sweden. We focus on experiences at the Unit for Personality Disorders, Sahlgrenska University Hospital in Gothenburg, using clinician reports and organizational observations to assess how the new framework influences routine care.

Early observations highlight practical challenges in integrating severity-based assessment into diagnostic workflows, treatment planning, and multidisciplinary collaboration. Organizational factors—including aligning level of care with severity, establishing a shared clinical language, and coordinating planning across specialties—emerge as important for facilitating implementation. Clinicians report potential benefits of the stratified approach, such as more nuanced clinical descriptions, improved communication, and individualized care planning, alongside ongoing hurdles in translating the model into practice within existing organizational structures.

These initial findings provide insight into the feasibility and implications of ICD-11 adoption in Sweden. They illustrate both the opportunities and practical challenges of implementing a dimensional model for PD, offering lessons relevant to other European and international settings.

**Disclosure of Interest:**

None Declared

